# Impaired germ cell development due to compromised cell cycle progression in Skp2-deficient mice

**DOI:** 10.1186/1747-1028-1-4

**Published:** 2006-04-07

**Authors:** Abbas Fotovati, Keiko Nakayama, Keiichi I Nakayama

**Affiliations:** 1 Department of Molecular and Cellular Biology, Medical Institute of Bioregulation, Kyushu University, Fukuoka, Fukuoka 812-8582, Japan; 2 CREST, Japan Science and Technology Agency, Kawaguchi, Saitama 332-0012, Japan; 3 Division of Developmental Genetics, Center for Translational and Advanced Animal Research on Human Diseases, Tohoku University School of Medicine, Sendai, Miyagi 980-8575, Japan

## Abstract

**Background:**

The gonads are responsible for the production of germ cells through both mitosis and meiosis. Skp2 is the receptor subunit of an SCF-type ubiquitin ligase and is a major regulator of the progression of cells into S phase of the cell cycle, which it promotes by mediating the ubiquitin-dependent degradation of p27, an inhibitor of cell proliferation. However, the role of the Skp2-p27 pathway in germ cell development remains elusive.

**Results:**

We now show that disruption of *Skp2 *in mice results in a marked impairment in the fertility of males, with the phenotypes resembling Sertoli cell-only syndrome in men. Testes of *Skp2*^-/- ^mice manifested pronounced germ cell hypoplasia accompanied by massive apoptosis in spermatogenic cells. Flow cytometry revealed an increased prevalence of polyploidy in spermatozoa, suggesting that the aneuploidy of these cells is responsible for the induction of apoptosis. Disruption of the *p27 *gene of *Skp2*^-/- ^mice restored germ cell development, indicating that the testicular hypoplasia of *Skp2*^-/- ^animals is attributable to the antiproliferative effect of p27 accumulation.

**Conclusion:**

Our results thus suggest that compromised cell cycle progression caused by the accumulation of p27 results in aneuploidy and the induction of apoptosis in gonadal cells of *Skp2*^-/- ^mice. The consequent reduction in the number of mature gametes accounts for the decreased fertility of these animals. These findings reinforce the importance of the Skp2-p27 pathway in cell cycle regulation and in germ cell development.

## Background

Infertility affects 10 to 15% of couples with up to half of fertility problems having a genetic etiology [1]. A major type of infertility is characterized by impaired production of germ cells. Germ cell development begins with the appearance of primordial germ cells at the early stage of embryogenesis. These cells migrate to the genital ridge, where they proliferate extensively by mitosis in both male and female embryos to establish the original pool of germ cells. The germ cells then enter a state of divisional arrest and remain in this state until sexual maturity, when they complete their developmental process by undergoing spermatogenesis in males and folliculogenesis in females. Such gametogenesis is highly sensitive to deficiency of various contributing factors. Targeted disruption of several genes in mice has thus defined key roles for many extracellular and intracellular signaling proteins in germ cell development and reproductive physiology [2-5].

Germ cells undergo both mitosis and meiosis during their development. Progression through the cell cycle requires the activity of two major ubiquitin ligase complexes, the Skp1-cullin-F-box protein (SCF) complex and the anaphase-promoting complex (APC) or cyclosome [6,7]. The F-box protein component of the SCF complex is variable, binds to Skp1 through its F-box motif, and is the subunit responsible for substrate recognition [8]. The F-box protein Skp2 plays an important role in progression of S phase of the cell cycle by contributing to the ubiquitin-dependent degradation of p27, a major inhibitor of proliferation in mammalian cells [6,9-11]. Skp2 begins to accumulate in late G_1 _phase of the cell cycle and its abundance is maximal during S and G_2 _phases [12-14]. We have previously generated mice that lack Skp2 and shown that the levels of p27 and of various other regulators of the cell cycle are increased in the cells of these animals [6,15-18]. Although *Skp2*^-/- ^mice are viable, their somatic cells contain markedly enlarged nuclei and manifest both polyploidy and multiple centrosomes [6]. Such defects are not apparent in *Skp2*^-/-^*;p27*^-/- ^mice, suggesting that they are largely the result of the abnormal accumulation of p27 in the *Skp2*^-/- ^animals [14,19].

We noticed that the fertility of male *Skp2*^-/- ^mice was reduced. We now show that, unlike other organs of these animals, the testes exhibit massive apoptosis, resulting in the loss of gametes. These characteristics were not apparent in *Skp2*^-/-^*;p27*^-/- ^mice, suggesting that accumulation of p27 accounts for the defect in germ cell development in the *Skp2*^-/- ^animals. These results indicate that regulation of cell cycle progression by the Skp2-p27 pathway is critical for germ cell development in both males and females.

## Results

### Reduced male fertility of Skp2-deficient mice

**Figure 1:**
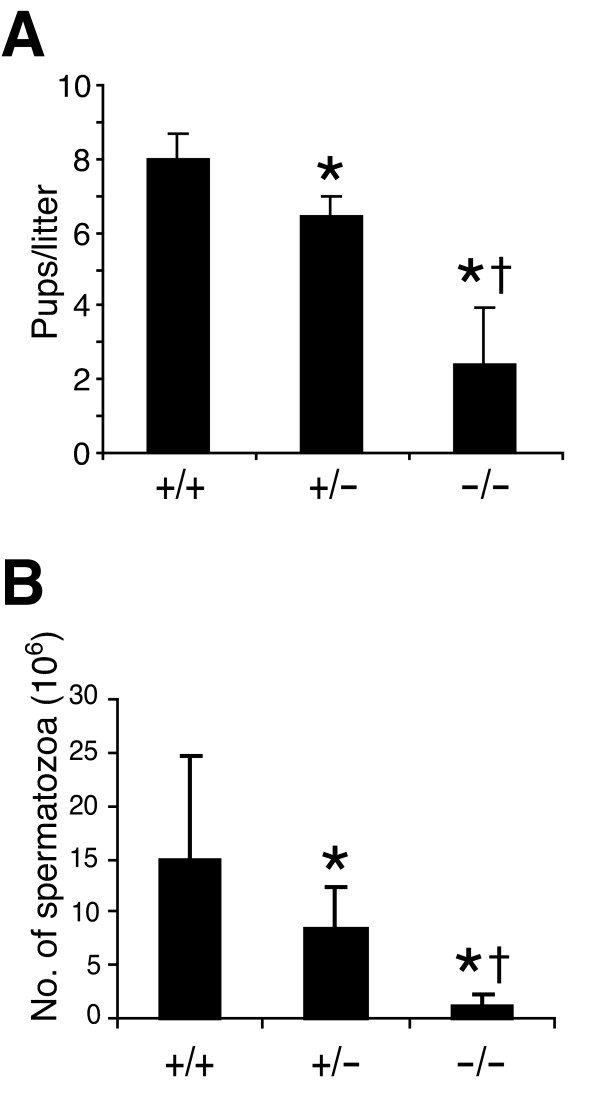
**Fertility and germ cell production in Skp2-deficient male mice. **(A) Fertility rate of male wild-type and mutant mice (2 to 4 months of age) as reflected by litter size when crossed with control C57BL/6 mice. Data are means ± SD for five animals per genotype. *P < 0.05 versus *Skp2*^+/+^, †*P *< 0.05 versus *Skp2*^+/-^. (B) Germ cell production in male wild-type and mutant mice. Epididymal sperm in 2-month-old mice were counted. Data are means ± SD for 8–12 mice per genotype. *P < 0.05 versus *Skp2*^+/+^, †*P *< 0.05 versus *Skp2*^+/-^.

The effect of Skp2 deficiency on the male fertility was examined separately by crossing with wild-type C57BL/6 mice. Mating of *Skp2*^-/- ^male mice with wild type pairs revealed a marked decrease in fertility, as evidenced by reduced litter size, compared with that of *Skp2*^+/+ ^pairs or *Skp2*^+/- ^pairs (Fig. 1A). The fertility of *Skp2*+/- pairs was also lower than that of wild-type pairs.

Epididymal spermatozoa were enumerated for evaluation of germ cell production in adult animals. Male *Skp2*^-/- ^mice exhibited a markedly reduced number of spermatozoa (azoospermia in some animals) compared with wild-type or heterozygous males (Fig. 1B). Furthermore, a substantial number of abnormally large cells and degenerative bodies was apparent in the semen of *Skp2*^-/- ^males (see Fig. 4D). The number of motile spermatzoa achieved >50% of the value for wild-type males in only one of 12*Skp2*^-/- ^males examined.

### Testicular atrophy in Skp2-deficient mice

**Figure 2:**
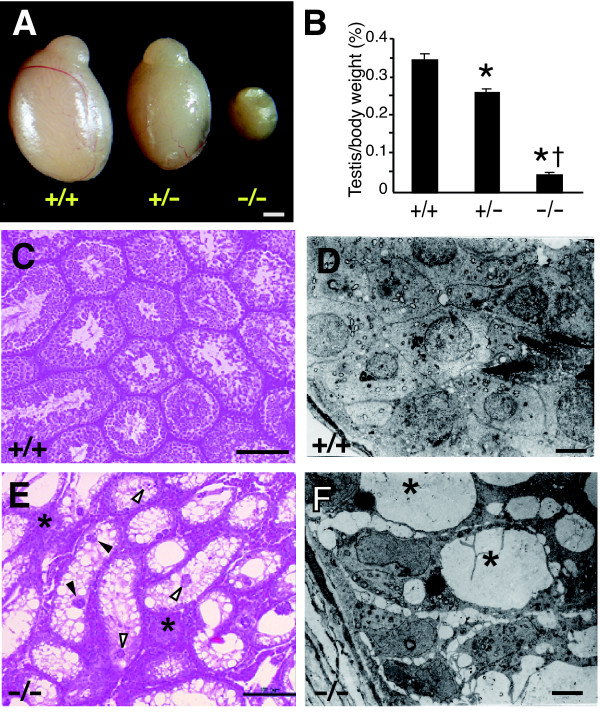
**Testicular morphology of Skp2-deficient mice. **(A) Macroscopic comparison of the testes of *Skp2*^+/+^, *Skp2*^+/-^, and *Skp2*^-/- ^mice at 2 months of age. Scale bar, 1 mm. *B*, Ratio of the weight of testes to body weight in male mice of the three genotypes at 2 months of age. Data are means ± SD for 6–8 animals of each genotype. *P < 0.05 versus *Skp2*^+/+^, †*P *< 0.05 versus *Skp2*^+/-^. (C-F) Representative light (C, E) and electron (D, F) micrographs of testicular sections of wild-type (C, D) and Skp2^-/- ^(E, F) males at 2–4 months of age. Note the pronounced loss of spermatogenic cells (asterisks in F), leaving only Sertoli cells, and the hyperplasia of the interstitial cellular population (asterisks in E) apparent adjacent to severely degenerated tubules in *Skp2*^-/- ^testis. Degenerating spermatocytes and clusters of round or elongated spermatids had detached from the seminiferous epithelium and been sloughed off into the tubule lumen of *Skp2*^-/- ^males (open arrowheads in E). Multinucleated giant spermatogenic cells were also present throughout the seminiferous epithelium of *Skp2*^-/- ^testis (closed arrowheads in E). Scale bars, 100 μm (C, E) or 5 μm (D, F).

The testes and accessory reproductive organs of *Skp2*^+/+^, *Skp2*^+/-^, and *Skp2*^-/- ^mice were further examined for histopathologic abnormalities that might contribute to the reduced fertility of the mutant animals. The testes of *Skp2*^-/- ^males were markedly hypotrophic and hypoplastic compared with those of *Skp2*^+/+ ^or *Skp2*^+/- ^animals (Fig. 2A). Given that the body size of *Skp2*^-/- ^mice is smaller than that of wild-type mice, we normalized testis weight by body weight for each animal; the normalized testis weight was still greatly reduced for *Skp2*^-/- ^mice compared with that for *Skp2*^+/+ ^or *Skp2*^+/- ^animals (Fig. 2B). No gross morphological abnormalities were apparent in accessory reproductive glands of *Skp2*^-/- ^males (data not shown).

Light and electron microscopic examination of the testes of *Skp2*^-/- ^mice revealed a progressive loss of spermatogonia with age, resulting in a marked depletion of germ cells in most seminiferous tubules (Fig. 2E, F) in comparison with wild-type testes (Fig. 2C, D). In advanced stages of germ cell loss, postmeiotic cells were rarely observed in seminiferous tubules of *Skp2*^-/- ^males, with only Sertoli cells remaining. This phenotype is similar to that of Sertoli cell-only syndrome in humans. Ultrastructural analysis revealed the presence of numerous vacuolated Sertoli cells, resulting from germ cell depletion, in the tubule epithelium of such *Skp2*^-/- ^males (Fig. 2F). Degenerating spermatocytes and clusters of round or elongated spermatids had detached from the seminiferous epithelium and been sloughed off into the tubule lumen (Fig. 2E). Multinucleated giant spermatogenic cells, containing from two to five or more nuclei, were also present throughout the seminiferous epithelium (Fig. 2E). The number and morphology of Sertoli cells in *Skp2*^-/- ^mice were otherwise similar to those in wild-type animals, whereas interstitial cellularity was increased, especially for Leydig cells, in the mutant males (Fig. 2E). The profile of spermatogenesis in most seminiferous tubules of *Skp2*^+/- ^mice was similar to that in wild-type animals, although detachment of spermatogenic cells was apparent in a few tubules of the heterozygotes (data not shown).

### Increased apoptosis in the gonads of Skp2-deficient mice

**Figure 3:**
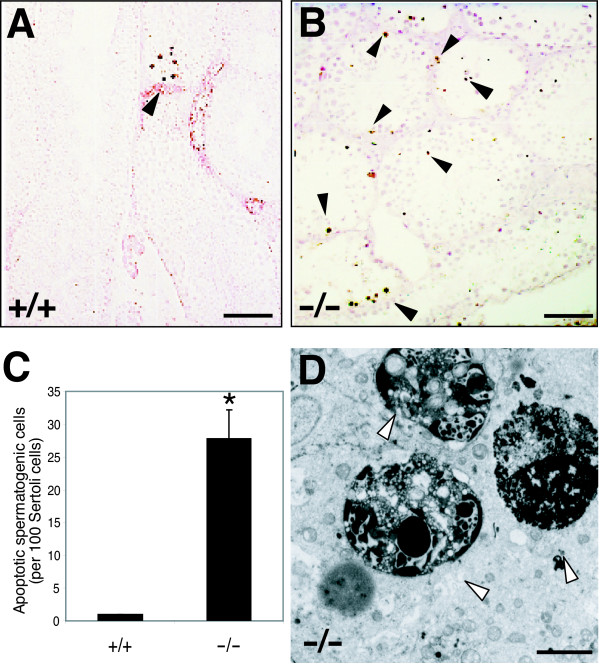
**Increased level of apoptosis in the gonads of *Skp2*^-/- ^mice. **(A, B) TUNEL staining of testicular sections of *Skp2*^+/+ ^(A) or *Skp2*^-/- ^(B) mice at 4 months of age. Arrowheads indicate apoptotic cells. Scale bars, 100 μm. (C) The number of apoptotic spermatogenic cells per 100 Sertoli cells. *P < 0.05 versus *Skp2*^+/+^. (D) Ultrastructural image of typical apoptotic figures (arrowheads) at late postmeiotic stages of spermatogenesis in a *Skp2*^-/- ^mouse at 4 months of age. Scale bar, 5 μm.

Cultured embryonic fibroblasts derived from *Skp2*^-/- ^mice manifest an increased prevalence of apoptosis compared with those derived from wild-type animals [6]. We therefore performed the TUNEL assay to determine whether apoptosis contributes to the progressive loss of germ cells in *Skp2*^-/- ^mice. Only a few spermatogenic cells of wild-type males, usually those at the spermatogonial stage, were found to be apoptotic (Fig. 3A). In contrast, a large proportion of spermatogenic cells, at almost all stages of spermatogenesis, was apoptotic in *Skp2*^-/- ^males (Fig. 3B). Statistical analysis confirmed that the ratio of apoptotic cells was significantly increased in *Skp2*^-/- ^mice (Fig. 3C). Electron microscopy also revealed the marked increase in the prevalence of apoptosis among spermatogenic cells, including those at the postmeiotic stage, in *Skp2*^-/- ^males (Fig. 3D). The level of apoptosis among supporting cells, including Sertoli, Leydig, and other interstitial cells, did not differ substantially between *Skp2*^-/- ^and wild-type males.

### Polyploidy and cellular dysmorphism of germ cells of Skp2-deficient mice

**Figure 4:**
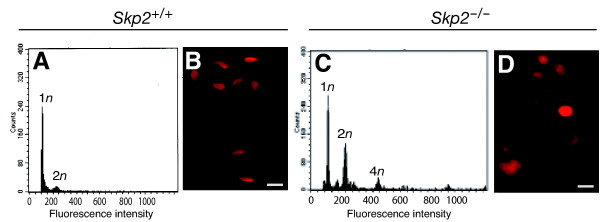
**Polyploidy of epididymal sperm and ovarian granulosa cells of *Skp2*^-/- ^mice. **(A, C) Flow cytometric analysis of the DNA content of seminal cells isolated from wild-type (A) and *Skp2*^-/- ^(C) males at 4 months of age. (B, D) Nuclear morphology of seminal cells isolated from wild-type (B) and *Skp2*^-/- ^(D) mice and stained with propidium iodide. Scale bars, 50 μm.

Isolated seminal cells were subjected to analysis of DNA content. Seminal cells from wild-type mice contained a majority of haploid (1*n*) cells, corresponding to normal mature spermatozoa (Fig. 4A). However, three out of seven (43%) *Skp2*^-/- ^males examined manifested a substantial number of polyploid cells in their semen (Fig. 4C); the proportion of haploid cells was thus reduced and that of diploid (2*n*), tetraploid (4*n*), and >4*n *cells was increased. Examination of the nuclear morphology of seminal cells by propidium iodide staining revealed a majority of laterally flattened, typically curved, falciform, hock-headed spermatozoa in wild-type males (Fig. 4B). In contrast, the seminal cells of *Skp2*^-/- ^males, in addition to some normally shaped haploid spermatozoa, contained a large number of cells with an abnormal nuclear size and shape (Fig. 4D).

### Effects of p27 accumulation in Skp2-defcient mice

**Figure 5:**
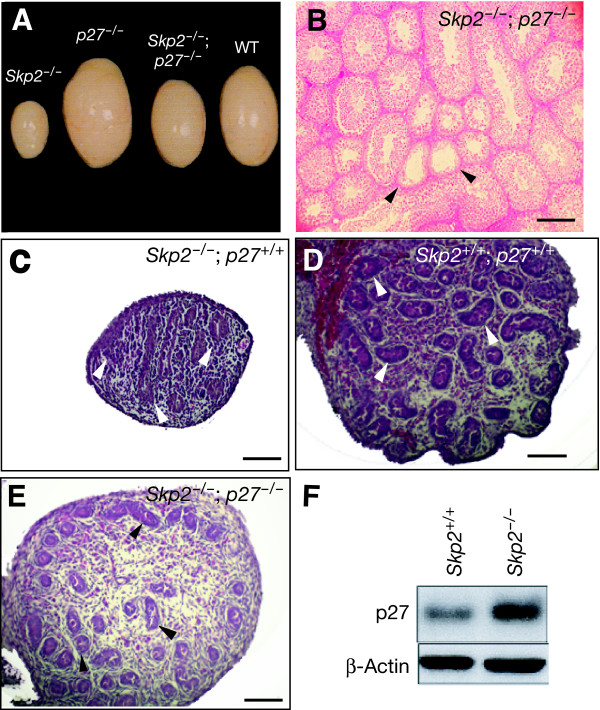
**Role of p27 accumulation in the gonadal hypoplasia of Skp2-deficient mice. **(A) Macroscopic comparison of the testes of wild-type, *Skp2*^-/-^, *p27*^-/-^, and *Skp2*^-/-^*;p27*^-/- ^mice. (B) Histology of the testis of *Skp2*^-/-^*;p27*^-/- ^mice (4 months of age) showing pronounced recovery of germ cell production compared with that apparent in parental *Skp2*^-/- ^mice. Scale bars, 100 μm. Arrowheads in B indicate seminiferous tubules with a deficiency of germ cells. (C-E) Histology of embryonic testis (15.5 days postcoitum). Severe deficiency of gonocytes (arrowheads) was evident in *Skp2*^-/- ^embryos (C) compared with wild-type embryos (D). However, the gonocyte population had recovered substantially in *Skp2*^-/-^;*p27*^-/- ^embryos (E). Scale bars, 100 μm. (F) Immunoblot analysis of lysates of the testes of *Skp2*^-/- ^or wild-type embryos (15.5 days postcoitum) with antibodies to p27 and to β-actin (loading control).

To examine the possible role of p27 in the impaired fertility of *Skp2*^-/- ^mice, we analyzed *Skp2*^-/-^*;p27*^-/- ^double-mutant animals. The gonads of the double-mutant mice showed a general reversal of the marked hypoplasia apparent in parental *Skp2*^-/- ^mice (Fig. 5A). Disruption of the *p27 *gene thus largely restored the pool of germ cells in both testis (Fig. 5B). A small number of germ cell-depleted seminiferous tubules was still apparent in double-mutant males, however. The fertility rate of double-mutant males (n = 6) when crossed with C57BL/6 females was also increased to 5.66 ± 1.03 pups per litter (see Fig. 1A). The double-mutant females were completely sterile, however, which is a characteristic of p27 deficiency [20].

The normalization of the reproductive systems of the double-mutant mice was apparent from early stages of germ cell development, especially in male animals. Although there was a substantial reduction in the number of gonocyte nests in the testes of *Skp2*^-/- ^embryos (Fig. 5C), the gonocyte reserves in double-mutant embryos (Fig. 5E) were similar to those in wild-type embryos (Fig. 5D). In addition, the amount of p27 in the embryonic testis at the early stage of germ cell development (15.5 days postcoitum) was markedly increased in *Skp2*^-/- ^mice compared with wild-type animals (Fig. 5F).

## Discussion

We have shown that male *Skp2*^-/- ^mice manifest a markedly reduced fertility. The gonads of male mutant mice exhibited a pronounced hypoplasia that was independent of the reduced body size of these animals. This gonadal hypoplasia was likely attributable to the lack of Skp2-dependent degradation of p27 by the proteasome [14,19]. Indeed, we have now shown that p27 accumulates in the testis of *Skp2*^-/- ^embryos at the early stage of gonocyte development. An important function of p27 is to restrain progression of the cell cycle, and this protein accumulates in response to many antiproliferative signals [21]. We and others previously showed that disruption of the *p27 *gene removes this brake and results in uncontrolled cell proliferation and hyperplasia of most organs, especially the testis and ovary [20,22,23]. In contrast, the accumulation of p27 in *Skp2*^-/- ^mice likely exerts an antiproliferative effect on embryonic germ cells, resulting in gonadal hypoplasia. This notion was supported by the observation that germ cell production was restored from an early stage of gonocyte development in *Skp2*^-/-^*;p27*^-/- ^double-mutant mice.

The marked depletion of spermatogenic cells apparent in adult *Skp2*^-/- ^males is reminiscent of that in infertile men with Sertoli cell-only syndrome [24]. The mutant male mice manifest hyperplasia of Leydig and other interstitial cells adjacent to the germ cell-depleted seminiferous tubules. A similar phenotype has also been described in human testicular disorders characterized by germ cell depletion and is thought to be a histological marker of testicular failure in men [25]. The accelerated depletion of germ cells in adult *Skp2*^-/- ^mice appears to be mediated by apoptosis, which is responsible for the removal of cells with abnormalities, such as an incorrect DNA content [26,27]. Such physiological apoptosis is a normal feature of both testis [28] and ovary, in the latter of which atretic follicles are prevented by apoptosis from achieving the final steps of follicular growth [28-30]. Adult *Skp2*^-/- ^mice manifested a greatly increased frequency of apoptosis during gametogenesis, however. In the testis, cells at various stages of spermatogenesis were found to be apoptotic, resulting in germ cell depletion.

The increased level of apoptosis apparent in the gonads of *Skp2*^-/- ^mice might be attributable to polyploidy, which was previously detected in somatic organs, including the liver, kidneys, and lungs, of these animals without any evident accompanying functional defects [6,14]. In general, abnormalities of nuclear DNA content during gametogenesis disturb the development of any resulting zygote, leading to infertility or embryo loss due to aneuploidy [31,32]. We now show that both germ cells and their supporting cells of *Skp2*^-/- ^mice are affected by polyploidy. In male mutant animals, polyploidy of spermatozoa likely contributed to the morphological abnormalities of these cells, given that morphologically abnormal (such as macrocephalic) spermatozoa have been shown to be polyploid both in mice [33] and in infertile men [34-38]. Polyploidy and consequent apoptosis are also likely to be responsible for detachment of spermatogonia and spermatocytes from the seminiferous epithelium and their formation of giant multinucleated structures in the tubules of *Skp2*^-/- ^males. Similar structures have been shown to represent syncytia of degenerating spermatids [39-42]. Aneuploidy has also been proposed to underlie the induction of spermatogenic cell apoptosis and the development of Sertoli cell-only syndrome in men [43].

The number of ova recoverable from *Skp2*^-/- ^female mice was too small to determine the presence of aneuploidy (data not shown). However, the high prevalence of apoptosis among ovarian granulosa cells was accompanied by an increased frequency of polyploidy in the mutant animals. Given the important role of granulosa cells in maintaining the female germ cells [44], follicles with apoptotic granulosa cells likely fail to progress to later stages of development and eventually undergo follicular atresia.

Impaired progression of the cell cycle caused by the absence of Skp2, an important controller of S phase, is likely responsible for the development of polyploidy in *Skp2*^-/- ^mice. Various chemical agents such as colchicines and vinblastine similarly affect both mitotic and meiotic cell division [45] and induce cell cycle delays in both somatic [46] and germ [47] cells, resulting in aneuploidy. Some of these chemicals delay progression of S or G_2 _phases of the cell cycle and thereby prolong cell cycle time [48,49]. Given that the abundance of Skp2 is maximal during S and G_2 _phases [12-14], it might be expected that its absence in these phases would lead to aneuploidy through a similar mechanism. Aneuploidy may also develop in the germ cells of *Skp2*^-/- ^mice by a mechanism similar to that operative in somatic cells of these animals; that is, endoreplication caused by the accumulation of p27 [14]. Indeed, the lack of p27 degradation during G_2 _phase in S*kp2*^-/- ^cells may result in suppression of Cdc2 activity and consequent inhibition of entry into M phase [14].

p27 antagonizes the activity of not only Cdc2, but also Cdk2 and Cdk4. Interestingly, mutant mice that lack the activity of Cdk2 or Cdk4 also exhibit the abnormalities in the gonadal development. *Cdk2*^-/- ^males and females are sterile with a severe atrophy of the gonads [50,51]. Cyclin E2-deficient males displayed reduced fertility, with approximately 50% of males being sterile [52]. *Cyclin E2*^-/- ^males displayed reduced testicular size and greatly reduced sperm counts, as compared with wild-type littermates. Cyclin E2-deficient females develop normally and are fully fertile. *Cdk4*^-/- ^mice showed reproductive dysfunction associated with hypoplastic seminiferous tubules in the testis and perturbed corpus luteum formation in the ovary [53,54]. Furthermore, cyclin D2-deficient females are sterile owing to the inability of ovarian granulosa cells to proliferate normally in response to follicle-stimulating hormone (FSH), whereas mutant males display hypoplastic testes [55]. Overall, these data suggest that Skp2-dependent control of p27 abundance plays a critical role in the regulation of the activity of Cdk2 and Cdk4, which is particularly important for the normal gonadal development.

## Conclusion

Our results suggest that Skp2, as an important regulator of S phase of the cell cycle, plays a key role in establishment of the original pool of gametic cells by mitosis during early embryogenesis as well as in the proliferation and maturation of these cells at later stages of development. Our findings reinforce the importance both of cell cycle regulators in germ cell development and of impaired function of such factors in fertility problems.

## Methods

### Animals

Skp2-deficient mice were generated by homologous recombination in embryonic stem cells as described previously [6]. Sexually mature mice from 2 to 12 months of age were used for experimental procedures. Pairs of *Skp2*^+/-^*;p27*^+/- ^mice were mated to produce *Skp2*^-/-^*;p27*^-/- ^animals [14]. All animal studies conformed with the Kyushu University Animal Experimentation Act. For evaluation of fertility, pairs of *Skp2*^+/+^, *Skp2*^+/-^, or *Skp2*^-/- ^mice were housed together for 8 weeks and then separated. Cages were monitored daily for the presence of seminal plugs, and the number and size of litters were recorded.

### Histopathology

For light microscopic analysis, tissue samples were fixed in either Bouin's fixative or 4% paraformaldehyde, dehydrated, treated with xylene, embedded in paraffin, and sectioned at a thickness of 5 μm. After removal of paraffin, the sections were dehydrated, rehydrated, and stained with hematoxylin-eosin. They were then examined under a Nikon Eclipse E800 microscope with either Nomarski or phase-contrast optics; images were photographed with a Hamamatsu 3CCD digital camera (model 7780). For electron microscopy, samples were fixed in glutaraldehyde immediately after resection and were then embedded in resin. Sections with a thickness of 80 nm were collected on copper grids and counterstained with lead citrate and uranyl acetate. They were then observed with a JEOL (JEM 2000) electron microscope at a voltage of 80 kV.

### TUNEL assay

For detection of apoptotic cells, tissue fixed in 4% paraformaldehyde or Bouin's solution was processed for the TUNEL (terminal deoxyribonucleotidyl transferase-mediated dUTP-biotin nick-end labeling)^1 ^assay essentially as described previously [56]. Apoptotic cells were visualized with the chromogen 3,3'-diaminobenzidine tetrahydrochloride (Sigma, St. Louis, MO).

### Isolation of spermatozoa

Mature male mice were killed by neck dislocation and the vas deferens and caudal epididymis were immediately exposed through a lower abdominal incision, excised, and washed briefly in phosphate-buffered saline (PBS). The tissue was then incubated in PBS for 30 min at 37°C and briefly massaged to promote the active exit of sperm from the epididymis. The released spermatozoa were isolated by centrifugation at 200 × *g *for 20 min, washed three times with PBS, counted with a hemocytometer attached to a light microscope, and analyzed for DNA content and morphology.

### Flow cytometric analysis of DNA content

Isolated spermatozoa or granulosa cells (1 × 10^6^) were washed in PBS, resuspended in 200 μl of PBS, and fixed by the gradual addition of 800 μl of 100% ice-cold ethanol. The fixed cells were washed twice with PBS, resuspended in 1 ml of PBS, treated with RNase (40 μg/ml) for 10 min at room temperature, and then stained with propidium iodide (25 μg/ml). The DNA content of the cells was determined by flow cytometry with a FACSCaliber instrument and CellQuest software (Becton Dickinson, San Jose, CA). The morphology of propidium iodide-stained spermatozoa was also examined with a fluorescence microscope.

### Immunoblot analysis

Embryonic testes were excised under a dissecting microscope, homogenized, and lysed in the presence of protease inhibitors. The tissue lysate (30 μg of protein) was fractionated by SDS-polyacrylamide gel electrophoresis, and the separated proteins were transferred to a nitrocellulose membrane and subjected to immunoblot analysis with rabbit polyclonal antibodies to mouse p27 (Santa Cruz Biotechnology, Santa Cruz, CA) and horseradish peroxidase-conjugated secondary antibodies. Immune complexes were detected with enhanced chemiluminescence reagents (Amersham Biosciences, Little Chalfont, UK). Immunodetection of β-actin was performed as a control for protein loading.

### Statistical analysis

Data are presented as means ± SD. The significance of differences between means was determined by ANOVA test. A *P *value of <0.05 was considered statistically significant.

## Abbreviations

TUNEL: terminal deoxyribonucleotidyl transferase-mediated dUTP-biotin nick-end labeling, PBS: phosphate-buffered saline

## Authors' contributions

FA performed all analyses of germ cell development. KN and KIN generated *p27*^-/-^, *Skp2*^-/-^, and *Skp2*^-/-^*;p27*^-/- ^mice. KIN is the principal investigator who gave advice in designing the study and edited the manuscript. All authors read and approved the final manuscript.
